# Molecular Determinants of Mechanical Itch Sensitization in Chronic Itch

**DOI:** 10.3389/fnmol.2022.937890

**Published:** 2022-06-16

**Authors:** Hankyu Lee, Robert D. Graham, Diana Melikyan, Brennan Smith, Ehsan Mirzakhalili, Scott F. Lempka, Bo Duan

**Affiliations:** ^1^Department of Molecular, Cellular, and Developmental Biology, University of Michigan, Ann Arbor, MI, United States; ^2^Department of Biomedical Engineering, University of Michigan, Ann Arbor, MI, United States; ^3^Biointerfaces Institute, University of Michigan, Ann Arbor, MI, United States; ^4^Department of Anesthesiology, University of Michigan, Ann Arbor, MI, United States

**Keywords:** Ucn3, NPY, Nav1.6, spinal cord, chronic itch

## Abstract

Chronic itch is associated with sensitization of the somatosensory nervous system. Recent studies have identified the neural circuits transmitting acute itch; however, the mechanisms by which itch transforms into a pathological state remain largely unknown. We have previously shown that Aβ low-threshold mechanoreceptors, together with spinal urocortin 3-positive (Ucn3^+^) excitatory interneurons and neuropeptide Y-positive (NPY^+^) inhibitory interneurons, form a microcircuit that transmits and gates acute mechanical itch. Here, using whole-cell patch-clamp recordings, we observed increased excitability in spinal Ucn3^+^ neurons under chronic itch conditions. In contrast to Ucn3^+^ neurons, the excitability of spinal NPY^+^ neurons was largely reduced under chronic itch conditions. To explore the molecular mechanisms underlying sensitization of this microcircuit, we examined the mRNA expression levels of voltage-gated ion channels in recorded spinal Ucn3^+^ and NPY^+^ neurons by single-cell quantitative real-time PCR (qRT-PCR). We found that the expression levels of Nav1.6 and Cav2.3 channels were increased in spinal Ucn3^+^ neurons in chronic itch mice, while the expression level of SK3 channels was decreased. By contrast, the expression levels of Nav1.6 and BK channels were decreased in spinal NPY^+^ neurons in chronic itch mice. To determine the contribution of different ion channels in chronic itch sensitization, we then used a Markov Chain Monte Carlo method to parameterize a large number of biophysically distinct multicompartment models of Ucn3^+^ and NPY^+^ neurons. These models included explicit representations of the ion channels that we found to be up- or down-regulated under chronic itch conditions. Our models demonstrated that changes in Nav1.6 conductance are predominantly responsible for the changes in excitability of both Ucn3^+^ and NPY^+^ neurons during chronic itch pathogenesis. Furthermore, when simulating microcircuits of our Ucn3^+^ and NPY^+^ models, we found that reduced Nav1.6 conductance in NPY^+^ models played a major role in opening the itch gate under chronic itch conditions. However, changing SK, BK, or R-type calcium channel conductance had negligible effects on the sensitization of this circuit. Therefore, our results suggest that Nav1.6 channels may play an essential role in mechanical itch sensitization. The findings presented here may open a new avenue for developing pharmaceutical strategies to treat chronic itch.

## Introduction

Itch can be evoked in the skin directly by physical stimuli, such as innocuous mechanical stimuli, referred to as mechanical itch, or by chemical mediators, such as histamine, referred to as chemical itch (Duan et al., 2018; Lay and Dong, 2020). Itch can be an acute sensation or a chronic condition. Chronic itch, such as atopic dermatitis and a variety of systemic diseases, presents major clinical challenges. Like many chronic pain conditions, chronic itch conditions are commonly associated with the peripheral and central sensitization of itch-signaling pathways, which results in chemical and mechanical itch sensitization, as well as spontaneous itch (LaMotte et al., 2014; Cevikbas and Lerner, 2020). The neural circuits that transmit chemical itch have been well identified in the last decade (Bautista et al., 2014; LaMotte et al., 2014; Lay and Dong, 2020). However, the molecular and cellular mechanisms of mechanical itch remain largely unknown.

**FIGURE 1:**
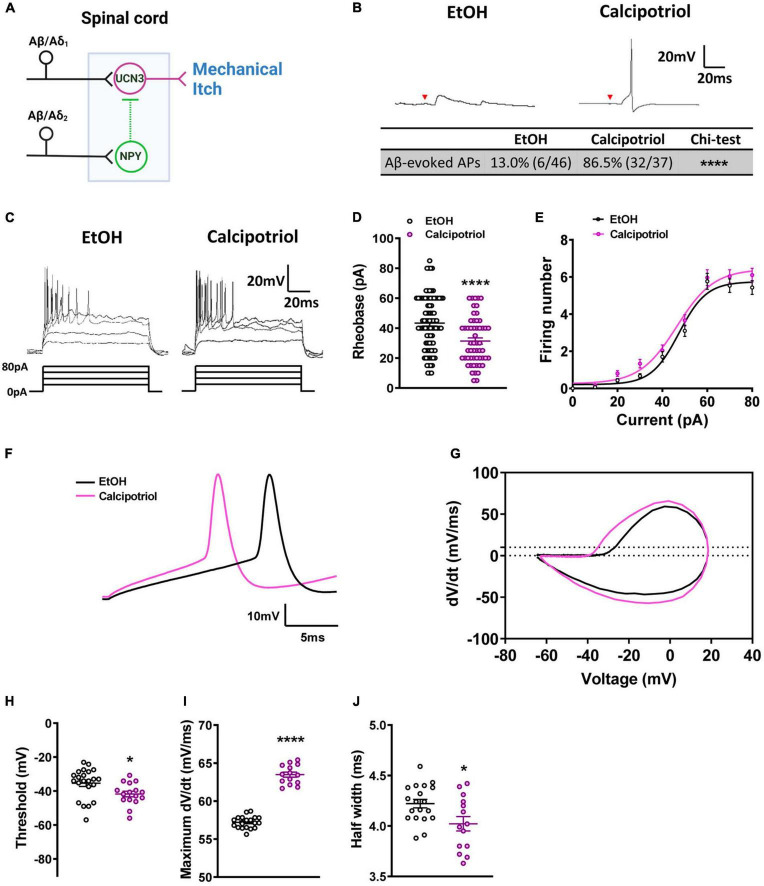
Action potential properties in Ucn3^+^ neurons. **(A)** Schematic diagram of the mechanical itch circuit in the dorsal spinal cord. **(B)** A typical trace of Aβ-evoked action potentials (Aβ-eAPs) in the recorded Ucn3^+^ neurons. These data included recordings from 46 neurons of EtOH-treated mice and 37 neurons of calcipotriol-treated Ucn3^+^ mice. **(C)** Firing properties of Ucn3^+^ neurons in EtOH- and calcipotriol-treated *Ucn3:Cre*; Ai14 mice. The majority of Ucn3^+^ neurons display an initial bursting firing pattern upon current injection. **(D)** Average AP rheobase in control and chronic itch mice. Control, *n* = 88; chronic itch, *n* = 55. ****p < 0.0001. Student’s unpaired *t*-test. **(E)** Average AP firing numbers responding to depolarizing currents in control and chronic itch mice. Control, *n* = 30; chronic itch, *n* = 30, no significant difference. ANOVA test. **(F)** Single AP induction upon current injection of Ucn3^+^ neurons in control and calcipotriol-treated chronic itch mice. **(G)** Phase plot (dV/dt vs. V) of the APs from **(F)**. **(H)** Average AP threshold in control and chronic itch mice. Control, *n* = 22; chronic itch, *n* = 14. *p < 0.05. Student’s unpaired *t*-test. **(I)** dV/dt during the transition from the resting membrane potential to the AP peak in control and chronic itch mice. Control, *n* = 19; chronic itch, *n* = 14. ****p < 0.0001. Student’s unpaired *t*-test. **(J)** Average AP half width in control and chronic itch mice. Control, *n* = 19; chronic itch, *n* = 14. *p < 0.05. Student’s unpaired *t*-test.

Recent studies have identified several key components of the neural circuit for mechanical itch. In the spinal cord, a subpopulation of excitatory interneurons expressing urocortin 3 (Ucn3^+^) in the dorsal horn presents a central node in this circuit for transmitting mechanical itch (Pan et al., 2019). This population receives peripheral inputs from Aβ-mechanoreceptors and is directly innervated by inhibitory interneurons expressing neuropeptide Y (NPY^+^) in the dorsal spinal cord (Bourane et al., 2015; Pan et al., 2019; Figure 1A). Under chronic itch conditions, this spinal microcircuit is sensitized for chronic persistent itch (Pan et al., 2019). In the periphery, mechanical itch is gated by Merkel cells and Aβ primary sensory fibers (Feng et al., 2018). A reduction in the number of Merkel cells and reduced expression of Piezo2 channels, as occur in aged mice or in dry skin conditions, are associated with mechanical itch sensitization (Feng et al., 2018).

Despite this progress, we know less about how the mechanical itch pathway is sensitized during the transition from acute to chronic itch conditions. In this study, we focused on the calcipotriol-induced atopic dermatitis mouse model and examined the altered expression of voltage-gated ion channels in both sensitized Ucn3^+^ neurons and desensitized NPY^+^ neurons in the dorsal horn. Computational modeling identified that reduced expression of Nav1.6 in NPY^+^ neurons could be a key mechanism underlying the sensitization of this spinal circuit in chronic itch at the single-neuron and network levels. Thus, our study identifies a novel contributor to atopic dermatitis that may represent an attractive target for future drug discovery efforts aimed at ameliorating this form of chronic itch.

## Materials and Methods

### Animals

We performed all animal experiments in accordance with protocols approved by the Institutional Animal Care and Use Committee at the University of Michigan following guidelines from the National Institutes of Health. We used both male and female mice for all experiments. We housed mice in groups at room temperature with *ad libitum* access to standard lab mouse pellet food and water on a 12 h light/12 h dark cycle. We used the following mouse lines in this study, namely, *Ucn3:Cre* (#032078-UCD, GENSAT), *NPY^Cre^* (#027851, JAX), and *Rosa26^LSL–tdTomato^* (Ai14, #007914, JAX). To label the *Ucn3:Cre*- or *NPY^Cre^*-derived neurons, we crossed *Ucn3:Cre* or *NPY^Cre^* mice with Ai14 reporter mice. We performed the atopic dermatitis model in mice as described previously (Pan et al., 2019). We dropped calcipotriol (2 nmol in 20 μl ethanol, Tocris Bioscience, United Kingdom) on the dorsal surface of the right hindpaw of adult mice (6–10 weeks) once daily for 7 days. We used vehicle (20 μl ethanol)-treated littermates as a control.

### Electrophysiology

#### Spinal Cord Slice Preparation

As described previously (Pan et al., 2019), we collected the parasagittal spinal cord slices attached with the full length of the dorsal root and dorsal root ganglion. We anesthetized mice deeply with isoflurane, decapitated, and rapidly removed the lumbar spinal cord and placed it in ice-cold modified artificial cerebrospinal fluid (ACSF) containing (in mM) 80 NaCl, 2.5 KCl, 1.25, NaH_2_PO_4_, 0.5 CaCl_2_, 3.5 MgCl_2_, 25 NaHCO_3_, 75 sucrose, 1.3 sodium ascorbate, and 3.0 sodium pyruvate, with pH at 7.4 and osmolality at 310–320 mOsm, oxygenated with 95% O_2_ and 5% of CO_2_. We used a VT1200s vibratome (Leica, Germany) to sagittally cut spinal cord slices (350–480 μm) attached with dorsal roots and dorsal root ganglia. We then incubated the slices for approximately 1 h at 33°C in oxygenated cutting solution containing (in mM) 125 NaCl, 2.5 KCl, 2 CaCl_2_, 1 MgCl_2_, 1.25 NaH_2_PO_4_, 26 NaHCO_3_, 25 D-glucose, 1.3 sodium ascorbate, and 3.0 sodium pyruvate, with pH at 7.2 and osmolality at 310–320 mOsm. We purchased all chemicals from MilliporeSigma (St. Louis, MO, United States).

#### Whole-Cell Patch-Clamp Recordings and Data Analysis

The whole-cell recording experiments have been described previously (Pan et al., 2019). The internal solution contained (in mM) 130 potassium gluconate, 5 KCl, 4 Na_2_ATP, 0.5 NaGTP, 20 HEPES, 0.5 EGTA, pH 7.28 with KOH, and measured osmolality at 310–320 mOsm. We acquired data using the pClamp 10.0 software (Molecular Devices, San Jose, CA, United States) with the MultiClamp 700B patch-clamp amplifier and Digidata 1550B (Molecular Devices, San Jose, CA, United States). We low-pass-filtered responses online at 2 kHz and digitized them at 5 kHz. We constructed phase plots from the first derivative of the somatic membrane potential (*dV/dt*) vs. the instantaneous somatic membrane potential.

### Dorsal Root Stimulation

As described previously (Pan et al., 2019), we evoked excitatory postsynaptic currents (eEPSCs) by the holding membrane potential at −70 mV, which minimized evoked inhibitory postsynaptic currents (eIPSCs). We recorded eIPSCs by holding the membrane potential at 0 mV, which minimized eEPSCs. We also used the transduction velocity (2.16–4.06 m/s) to determine monosynaptic Aβ inputs. We determined monosynaptic inputs for Aβ fibers by high-frequency stimulation at 20 Hz. We determined action potential (AP) firing patterns from current-clamp recordings at the resting membrane potential.

### Single-Cell Quantitative Real-Time PCR

We generated PCR-amplified cDNA libraries for single cells from individual spinal cord neurons after electrophysiological recording (SuperScript™ IV Single Cell cDNA PreAmp, Thermo, CA, United States). We combined 1 × reaction mix (Thermo, CA, United States), MgCl_2_ (2 mM), each deoxynucleotide (250 mM, Thermo, CA, United States), primers (0.25 mM), and 2.5 U SuperScript™ One-Step RT-PCR (Thermo, CA, United States) with 1 μl template cDNA. The quantitative real-time PCR (qRT-PCR) amplification procedure was as follows: an initial denaturation for 10 min at 94°C, 35 cycles of 30 s denaturation (94°C), 30 s annealing (55°C), and 2 min extension (72°C), and a final extension for 5 min at 72°C. The sequence of the primers is listed in Table 1. We determined the expression levels of mRNA by quantitative real-time PCR with Applied Biosystems™ 7500 Real-Time PCR Systems (Thermo, CA, United States).

**TABLE 1:** The sequences of primers used in quantitative real-time PCR (qRT-PCR).

Target Gene	Sequences		Reference sequence
Urocortin 3 (Ucn3)	F 20	CAGACAAGCCCAAAAGCGAC	NM_031250.5
	R 20	GCCTTGGCTCGCAAATTCTT	
Neuropeptide Y (Npy)	F 21	CTCGTGTGTTTGGGCATTCTG	NM_023456.3
	R 19	GTGTCGCAGAGCGGAGTAG	
Nav1.3 (Scn3a)	F 20	GCAGCATCTTCAGCTTTCGG	NM_006922
	R 20	TAAAGGGATGAGCGACAAA	
Nav1.6 (Scn8a)	F 20	GCAGCATCTTCAGCTTTCGG	NM_001077499.1
	R 17	AGGCCCGATGAGCGACA	
SK3 (Kcnn3)	F 22	TTATCCACCGTCATCCTGCTTG	NM_080466.2
	R 20	GCCCGAGATGGGGTATAGGA	
Kv4.2 (Kcnd2)	F 21	ATGAACCGGCCTTCATAAGCA	NM_019697.4
	R 20	GACTGTGACTTGATGGGCGA	
BK (Kcnma1)	F 20	GTGGGAAACACTGCACAACT	NM_001253358.1
	R 20	CAAGACCCCGATGCTGTCAT	
Cav2.3 (Cacna1e)	F 23	AGTGTGAAGAAGAACGCATCAGT	NM_009782.3
	R 20	ATGCAGAGGCAGAGTCCTTG	
Cav3.2 (Cacna1h)	F 20	CCTCACGTTGTTCCGAGTGT	NM_021415.4
	R 20	GTACCTTGGCTTTCCTGTGC	
GAPDH (Gapdh)	F 22	TGAAGGTCGGTGTGAACGAATT	XM_001473623.1
	R 22	GCTTTCTCCATGGTGGTGAAGA	

### Biophysical Models of Superficial Dorsal Horn Interneurons

We constructed all Ucn3^+^ and NPY^+^ multicompartment cable models with the NEURON simulation environment (v7.7) (Hines and Carnevale, 1997) within the Python programming language (Hines et al., 2009). We used a temperature of 23°C and an integration time step of 20 μs in each simulation. We implemented a previously published multicompartment cable model of a superficial dorsal horn interneuron (Melnick et al., 2004a,b) to model Ucn3^+^ and NPY^+^ neurons. The model utilized a simplified ball-and-stick morphology to accurately reproduce morphological and electrophysiological features of spinal cord interneurons (e.g., cell body size, equivalent dendrite impedance) without increasing computational demand. We implemented all morphological parameters described by Melnick et al. (2004b), with the addition of a myelinated axon with 41 nodes of Ranvier.

The original spinal interneuron model contained a voltage-gated sodium channel and a delayed-rectifier potassium channel that reproduced tonic- and adapting-firing behavior seen in some superficial dorsal horn interneurons (Melnick et al., 2004a,b). However, in this study, we aimed to determine which specific ion channels contribute to the biophysical features of chronic itch pathogenesis. Therefore, we removed the original ion channel models and instead included explicit representations of the voltage-gated sodium channel Nav1.6 (Hu et al., 2009), a delayed rectifier potassium channel (Gillies and Willshaw, 2006), an A-type potassium channel (Choi and Waxman, 2011), a small-conductance calcium-activated (SK) potassium channel (Gillies and Willshaw, 2006), an R-type voltage-gated calcium channel (Markaki et al., 2005), a big conductance calcium- and voltage-activated (BK) potassium channel (Gidon et al., 2020), and a linear leak conductance. To reduce dimensionality during model parameterization and computational demand during single-cell and network simulations, we expressed these ion channels only in the axon hillock. We modeled the dendritic and somatic compartments as passive cables with only a linear leak conductance and membrane capacitance. The 41 nodal compartments in the axon consisted of NEURON’s built-in Hodgkin-Huxley dynamics (i.e., the “hh” mechanism in NEURON) to allow AP propagation along the axon.

To generate populations of Ucn3^+^ and NPY^+^ model neurons with variable ion channel expression profiles, we implemented Goodman and Weare’s (2010) Affine-Invariant Markov Chain Monte Carlo method (MCMC) (Mackey et al., 2013) using the emcee Python package.^1^ MCMC methods have previously been used to simultaneously estimate the values of several ion current parameters in a biophysical neuron model (Adams et al., 2018). We extended this approach to generate *de novo* combinations of maximal ion channel conductances with which to parameterize our Ucn3^+^ and NPY^+^ model neurons. MCMC methods use Bayes’ theorem to estimate the posterior probabilities of a given set of parameter values describing a system based on experimental data and prior probabilities of each parameter’s value.

### Simulating the Mechanical Itch Pathway

We wanted to examine how changes in ion channel expression affected the output of the mechanical itch network, i.e., how changes in ion channel expression affected whether Ucn3^+^ neurons generated APs in response to physiological Aβ-fiber input (Pan et al., 2019). Therefore, in some simulations, we modeled synaptic connections between Ucn3^+^ and NPY^+^ model neurons and simulated how calcipotriol-induced changes in ion channel expression affected network behavior. We modeled all synaptic connections as alpha functions using NEURON’s built-in Exp2Syn mechanism. We modeled the inhibitory synapse from the last node of Ranvier in an NPY^+^ neuron onto the somata of Ucn3^+^ neurons with a 0.1 ms rise time, a 20 ms decay time constant, and a reversal potential of −75 mV (Zhang et al., 2014; Moraud et al., 2016). We modeled Aβ-fiber input as point processes onto the middle of the dendrite of both Ucn3^+^ and NPY^+^ neurons. We modeled the excitatory Aβ-fiber input using a rise time of 0.1 ms, a 5 ms decay constant, and a reversal potential of 0 mV (Zhang et al., 2014). The latency of monosynaptic input from Aβ-fibers onto Ucn3^+^ neurons is longer than the latency of Aβ-fiber input onto NPY^+^ neurons. Therefore, we modeled Aβ-fiber input onto Ucn3^+^ and NPY^+^ neurons with latencies of 4.0 and 2.2 ms based on spinal cord slice recordings, respectively. We set the conductance of all excitatory and inhibitory synapses such that they produced evoked postsynaptic currents of similar amplitude to previous experimental measurements (Pan et al., 2019).

### Statistical Analysis

Results are expressed as mean ± SEM. We performed statistical analysis in Prism (GraphPad). We used the unpaired Student’s *t*-test, chi-squared test, or Mann-Whitney *U* rank test to perform comparisons between two groups. We used the two-way ANOVA and Sidak *post-hoc* test for pairwise comparisons among more than two groups. We established statistical significance with a threshold of *p* < 0.05.

## Results

### Action Potential Properties Are Changed in Urocortin 3-Positive and Neuropeptide Y-Positive Neurons Under Chronic Itch Conditions

Mechanical itch sensitization and spontaneous itch are hallmarks of chronic itch (Duan et al., 2018). In our previous study, we found that the mechanical itch pathway in the spinal cord is sensitized under chronic itch conditions (Pan et al., 2019). However, it is unknown if there are changes in the excitability of Ucn3^+^ neurons. In this study, we first examined the excitability of Ucn3^+^ neurons under chronic itch conditions. We marked Ucn3^+^ neurons in the dorsal spinal cord by crossing *Ucn3:Cre* and Ai14 strains and then recorded tdTomato^+^ neurons in spinal cord slices in control and calcipotriol-treated chronic itch mice. We found that 13.0% (6/46) of Ucn3^+^ neurons were excited by Aβ stimulation in the vehicle (EtOH)-treated control group, while 86.5% (32/37) of Ucn3^+^ neurons were excited by Aβ stimulation in the calcipotriol-treated group (*p* < 0.0001, chi-squared test, Figure 1B), indicating that the mechanical itch microcircuit in the spinal cord is sensitized under chronic itch conditions. Next, we examined the AP properties of Ucn3^+^ neurons. The majority of Ucn3^+^ neurons exhibited initial bursting firing in response to depolarizing current pulses in both the control and chronic itch groups (Figure 1C). The minimum current injected to generate an AP (rheobase) was 27.7% lower in the calcipotriol-treated group (*n* = 55) compared to the control group (*n* = 88; *p* < 0.0001, unpaired Student’s *t*-test, Figure 1D). But the number of APs generated in response to depolarizing currents remained unchanged (Figure 1E). We then compared responses of Ucn3^+^ neurons to 60 pA current injection using phase plot analysis and plots of the membrane potential slope (*dV/dt*) vs. the membrane potential (Figures 1F,G). Moreover, we used a primary criterion of the membrane potential at a phase plot slope of 10 mV/ms to estimate AP threshold (Kress et al., 2008; Yu et al., 2008). The threshold for AP firing in the calcipotriol-treated group showed a significant decrease (−41.7 mV, *n* = 16) compared to the control group (−35.5 mV, *n* = 22, *p* < 0.05, unpaired Student’s *t*-test, Figure 1H). There was a significant increase (11%) in the maximum *dV/dt* when transitioning from the resting membrane potential to the peak of the AP (Figure 1I), and the half width of a single AP also showed a significant decrease (control: 4.2 ms, *n* = 19; calcipotriol: 4.0 ms, *n* = 14, *p* < 0.05, unpaired Student’s *t*-test, Figure 1J). The differences in the AP properties suggest an increased excitability of Ucn3^+^ neurons under chronic itch conditions.

**FIGURE 2:**
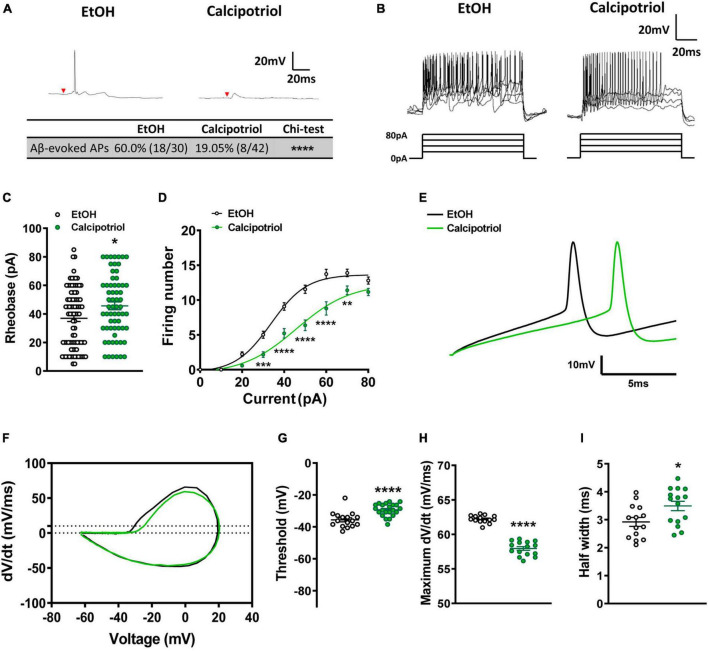
Action potential properties in NPY^+^ neurons. **(A)** A typical trace of Aβ-evoked action potentials (Aβ-eAPs) in the recorded NPY^+^ neurons. These data included recordings from 30 neurons of EtOH-treated mice and 42 neurons of calcipotriol-treated *NPY^Cre^*;Ai14 mice. **(B)** Firing properties of NPY^+^ neurons in EtOH and calcipotriol-treated *NPY^Cre^*;Ai14 mice. The majority of NPY^+^ neurons display a tonic firing pattern upon current injection. **(C)** Average AP rheobase in control and chronic itch mice. Control, *n* = 86; chronic itch, *n* = 63. *p < 0.05. Student’s unpaired *t*-test. **(D)** Average AP firing numbers responding to depolarizing currents in control and chronic itch mice. Control, *n* = 38; chronic itch, *n* = 44, **p < 0.01, ***p < 0.001, ****V < 0.0001. ANOVA test. **(E)** Single AP induction upon current injection in NPY^+^ neurons in control and calcipotriol-treated chronic itch mice. **(F)** Phase plot (dV/dt vs. V) of the AP from **(E)**. **(G)** Average AP threshold in control and chronic itch mice. Control, *n* = 15; chronic itch, *n* = 22. ****p < 0.0001. Student’s unpaired *t*-test. **(H)** dV/dt during the transition from the resting membrane potential to the AP peak in control and chronic itch mice. Control, *n* = 14; chronic itch, *n* = 15. ****p < 0.0001. Student’s unpaired *t*-test. **(I)** Average AP half width in control and chronic itch mice. Control, *n* = 14; chronic itch, *n* = 15. *p < 0.05. Student’s unpaired *t*-test.

Next, we examined whether the excitability of NPY^+^ neurons was altered under chronic itch conditions. We marked NPY^+^ neurons in the dorsal spinal cord by crossing *NPY^Cre^* and Ai14 strains and then recorded tdTomato^+^ neurons in spinal cord slices in control and calcipotriol-treated chronic itch mice. Interestingly, most NPY^+^ neurons were inactivated following Aβ stimulation in the calcipotriol-treated group (control: 60.0%; calcipotriol-treated: 19.0%, *p* < 0.0001, chi-squared test, Figure 2A), indicating that NPY^+^ inhibitory interneurons are silent for gating mechanical itch under chronic itch conditions. Next, we examined the AP properties of NPY^+^ neurons. The rheobase increased by 23.4 ± 2.2% in the calcipotriol-treated group (*n* = 63) compared to the control group (*n* = 86; *p* < 0.05, unpaired Student’s *t*-test, Figures 2B,C). Consequently, the number of APs activated in response to depolarizing currents of increasing intensities was largely reduced in the calcipotriol-treated group (Figure 2D). In the phase plot analysis (Figures 2E,F), there was a significant increase in AP threshold (control: −35.6 mV, *n* = 16; calcipotriol: −29.3 mV, *n* = 22, *p* < 0.0001, unpaired Student’s *t*-test, Figure 2G) and a decrease (6.8%) in the maximum *dV/dt* when transitioning from the resting membrane potential to the peak of the AP (Figure 2H), with a larger AP half-width in the calcipotriol-treated group (control: 2.9 ms, *n* = 14, calcipotriol: 3.5 ms, *n* = 15, *p* < 0.05, unpaired Student’s *t*-test, Figure 2I), suggesting a decreased excitability of NPY^+^ neurons under chronic itch conditions.

### Altered Expression of Voltage-Gated Ion Channels in Urocortin 3-Positive and Neuropeptide Y-Positive Neurons Under Chronic Itch Conditions

**FIGURE 3:**
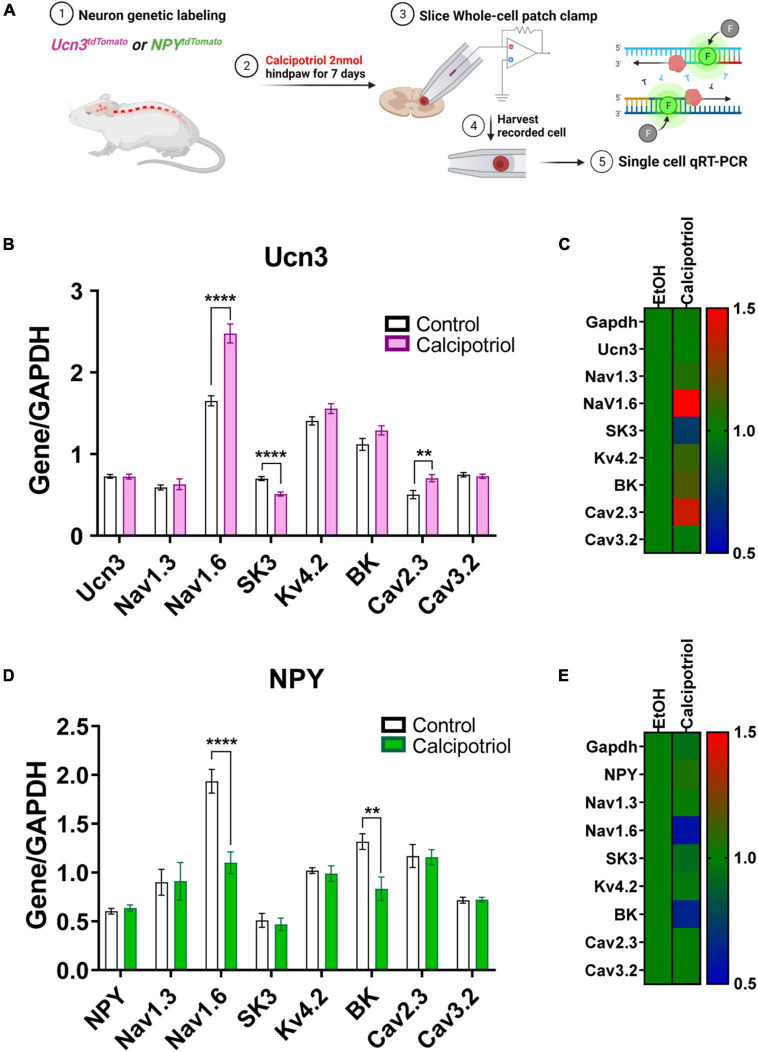
Single-cell quantitative real-time PCR (qRT-PCR) analysis of recorded Ucn3^+^ and NPY^+^ neurons. **(A)** Schematic diagram of the entire experimental method. **(B)** Single-cell qRT-PCR of normalized Ucn3^+^ neurons based on GAPDH in EtOH and calcipotriol-treated mice. Control, *n* = 14; chronic itch, *n* = 11. **p < 0.01, ****p < 0.0001. Student’s unpaired *t*-test. **(C)** The control gene was normalized to 1, and the relative change for each gene is expressed as a heat map. Control, *n* = 14; chronic itch, *n* = 11. **(D)** Single-cell qRT-PCR of normalized NPY^+^ neurons based on GAPDH in EtOH and calcipotriol-treated mice. Control, *n* = 23; chronic itch, *n* = 23. **p < 0.01, ****p < 0.0001. Student’s unpaired *t*-test. **(E)** The control gene was normalized to 1, and the relative change for each gene is expressed as a heat map. Control, *n* = 23; chronic itch, *n* = 23.

These changes in AP properties indicate that there may be a difference in ion channel expression in Ucn3^+^ or NPY^+^ neurons under chronic itch conditions. To examine altered expression of voltage-gated ion channels, we harvested the cells after whole-cell patch-clamp recording and tested mRNA expression levels of candidate genes through single-cell qRT-PCR (Figure 3A). Candidate genes included voltage-gated ion channels that are expressed in the dorsal spinal cord based on the Mouse Spinal Cord Atlas at Brainmap.org. We screened candidate genes *via* single-cell qPCR using recorded Ucn3^+^ and NPY^+^ neurons and then focused on the following genes identified in Ucn3^+^ or NPY^+^ neurons at relatively high levels, including Nav1.1 (*Scn1a*), Nav1.2 (*Scn2a*), Nav1.3 (*Scn3a*), Nav1.6 (*Scn8a*), Kv4.2 (*Kcnd2*), SK3 (*Kcnn3*), BK (*Kcnma1*), Cav2.3 (*Cacna1e*), and Cav3.2 (*Cacna1h*). We also tested *Gapdh* and *Ucn3* (or *Npy*) genes as controls. In Ucn3^+^ neurons, we found that the mRNA expression levels of Nav1.6 and Cav2.3 were upregulated in the calcipotriol-treated group, while the mRNA expression level of SK3 was downregulated in the calcipotriol-treated group (Figures 3B,C). In NPY^+^ neurons, we found that the mRNA expression levels of Nav1.6 and BK were downregulated in the calcipotriol-treated group. As comparison, the expression levels of other genes remained unchanged in the calcipotriol-treated group (Figures 3D,E).

### Model Population Characteristics

**FIGURE 4:**
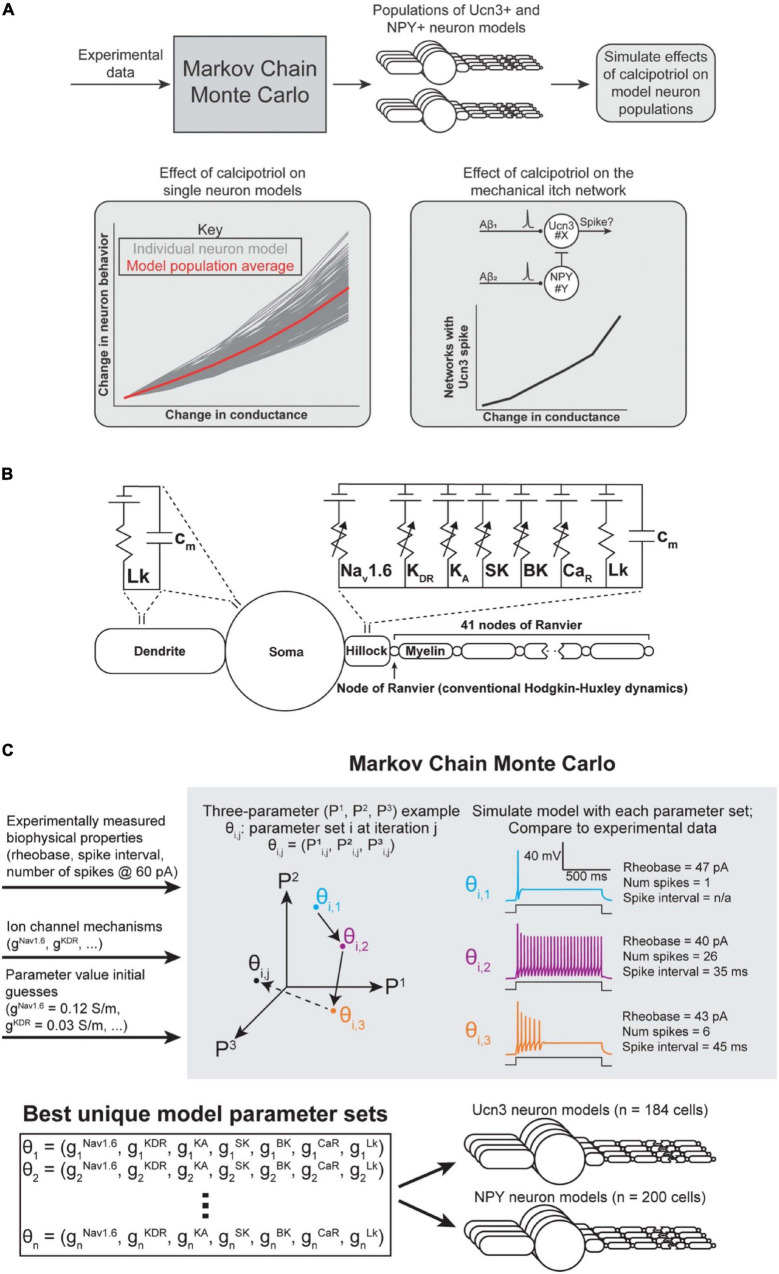
Spinal interneuron model and model population parameterization. **(A)** Flowchart of computational modeling methods. **(B)** Multicompartment model of a superficial dorsal horn interneuron expressing the ion channels found to be up- or down-regulated in response to calcipotriol. **(C)** Markov Chain Monte Carlo (MCMC) method for parameterizing populations of Ucn3^+^ and NPY^+^ model neurons. An example with three unknown parameters (P1, P2, P3) is shown for visualization purposes. Our implementation of the MCMC algorithm had seven unknown parameters (gNav1.6, gKDR, gKA, gSK, gBK, gCaR, gLk). The MCMC algorithm produced 184 unique models of Ucn3^+^ neurons and 200 unique models of NPY^+^ neurons.

As described above, we observed several concurrent changes in the AP properties and ion channel expression levels of Ucn3^+^ and NPY^+^ neurons under chronic itch conditions. We next aimed to determine which changes in ion channel expression were chiefly responsible for the changes that we observed in single-cell biophysical properties in calcipotriol-treated mice (Figure 4A). We employed a multicompartment cable modeling approach to investigate how changes in ion channel expression led to the biophysical changes (e.g., change in rheobase) observed during calcipotriol-induced chronic itch pathogenesis (Figure 4B). We utilized an MCMC algorithm to simulate and evaluate thousands of possible parameter sets (i.e., combinations of maximal ion channel conductances) on their ability to reproduce experimentally measured biophysical characteristics of Ucn3^+^ and NPY^+^ neurons (Figure 4C).

For each run of the MCMC algorithm, we simulated 400 individual Markov chains (i.e., walkers) of parameter sets (Figure 4C). We allowed each walker to modify and re-evaluate its parameter values 25 times per run (i.e., 25 iterations). We ensured sufficient exploration of the parameter space by running the MCMC algorithm four times for each cell type (i.e., Ucn3^+^ and NPY^+^ neurons), and injecting Gaussian noise in each walker’s initial guesses for each parameter value during each run. We chose uniform distributions constrained within physiological ranges (e.g., conductances cannot be negative) as priors for each parameter. This process resulted in thousands of possible parameter combinations that could parameterize a Ucn3^+^ or NPY^+^ model neuron. We removed parameter sets that poorly matched experimentally measured biophysical characteristics (e.g., rheobase) by excluding models above a score threshold (i.e., a normalized average distance between each metric greater than 0.6) from analysis.

**FIGURE 5:**
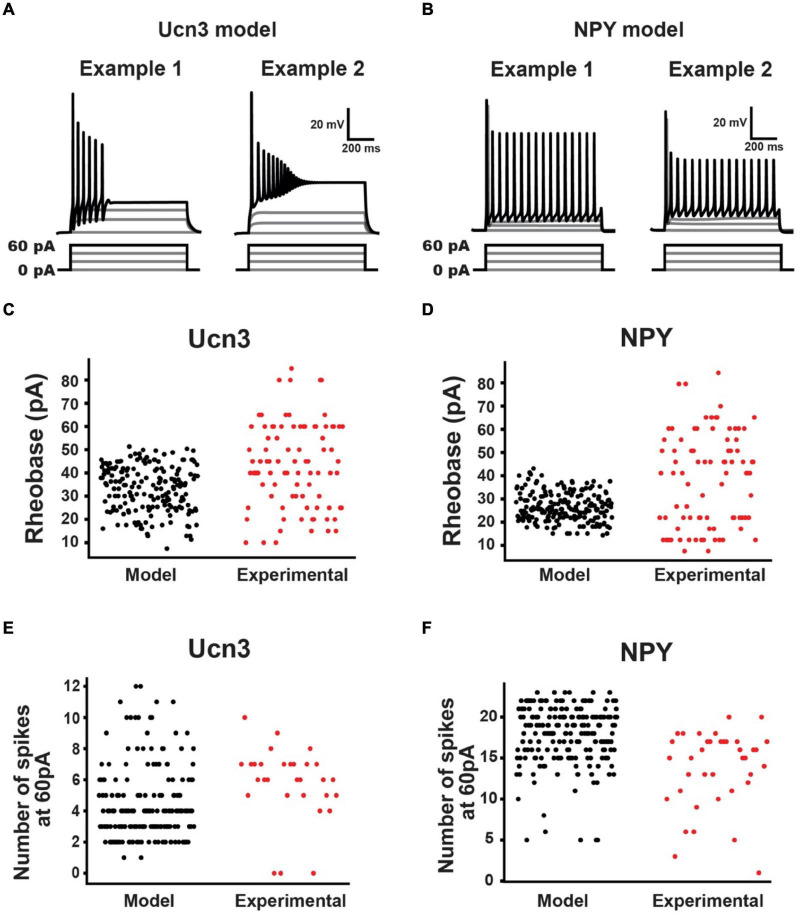
Urocortin 3-positive (Ucn3^+^) and NPY^+^ model neuron population characteristics. **(A)** Example Ucn3^+^ model neuron firing patterns during current injection. Ucn3^+^ model neurons demonstrated initial bursting patterns similar to our experimentally measured cells. **(B)** Example NPY^+^ model neuron firing patterns during current injection. NPY^+^ model neurons demonstrated tonic firing patterns similar to our experimentally measured cells. **(C)** Comparison of Ucn3^+^ model neuron rheobases (black) with experimentally measured Ucn3^+^ neuron rheobases (red). **(D)** Comparison of NPY^+^ model neuron rheobases (black) with experimentally measured NPY^+^ neuron rheobases (red). **(E)** Comparison of Ucn3^+^ model neuron number of spikes generated in response to 60 pA current injection (black) with those of experimentally measured Ucn3^+^ neurons (red). **(F)** Comparison of NPY^+^ model neuron number of spikes generated in response to 60 pA current injection (black) with those of experimentally measured NPY^+^ neurons (red).

The result of this process was 184 parameter sets that described Ucn3^+^ model neurons, and 200 parameter sets that described NPY^+^ model neurons (Figure 4C). These models produced current-clamp responses similar to those seen experimentally in Ucn3^+^ neurons (Figures 1B, 5A) and NPY^+^ neurons (Figures 2B, 5B). The distributions of the model populations’ biophysical properties (i.e., rheobase, number of spikes generated at 60 pA) resembled the distribution of biophysical properties measured from our experimental population (Figures 5C–F). Our model populations appear to resemble the more-excitable (lower rheobase) cells observed in our experimental population of neurons (Figures 5C,D). It is possible that mechanical itch circuits comprised of the most excitable population of each cell type may be most susceptible to chronic itch-induced changes. Therefore, we believe that our Ucn3^+^ and NPY^+^ model populations could provide valuable insight into the mechanisms of chronic itch pathogenesis.

### Effect of Ion Channel Conductance on Single-Cell Biophysical Properties

Next, we simulated experimentally measured changes in ion channel expression in each model neuron to determine which changes in ion channel expression were chiefly responsible for changes in single-cell excitability metrics (e.g., rheobase). We mimicked the changes in ion channel mRNA expression by systematically increasing or decreasing the conductance of the corresponding ion channel in each of our Ucn3^+^ and NPY^+^ models. We assumed that an increase in mRNA expression of a given channel likely corresponded to an increase in the number of those channels inserted into the neural membrane. Similarly, we assumed that a decrease in mRNA expression corresponded to a decrease in the number of channels inserted into the membrane. We further assumed that the single-channel conductance of a given ion channel was equal across all instances of that channel. Therefore, increasing or decreasing the maximal conductance of a given ion channel in our modeling framework mimics an increase or decrease in mRNA expression, respectively. We then calculated the biophysical properties of each cell model (e.g., rheobase) to determine how changes in ion channel expression may alter the excitability of neurons in the mechanical itch network.

**FIGURE 6:**
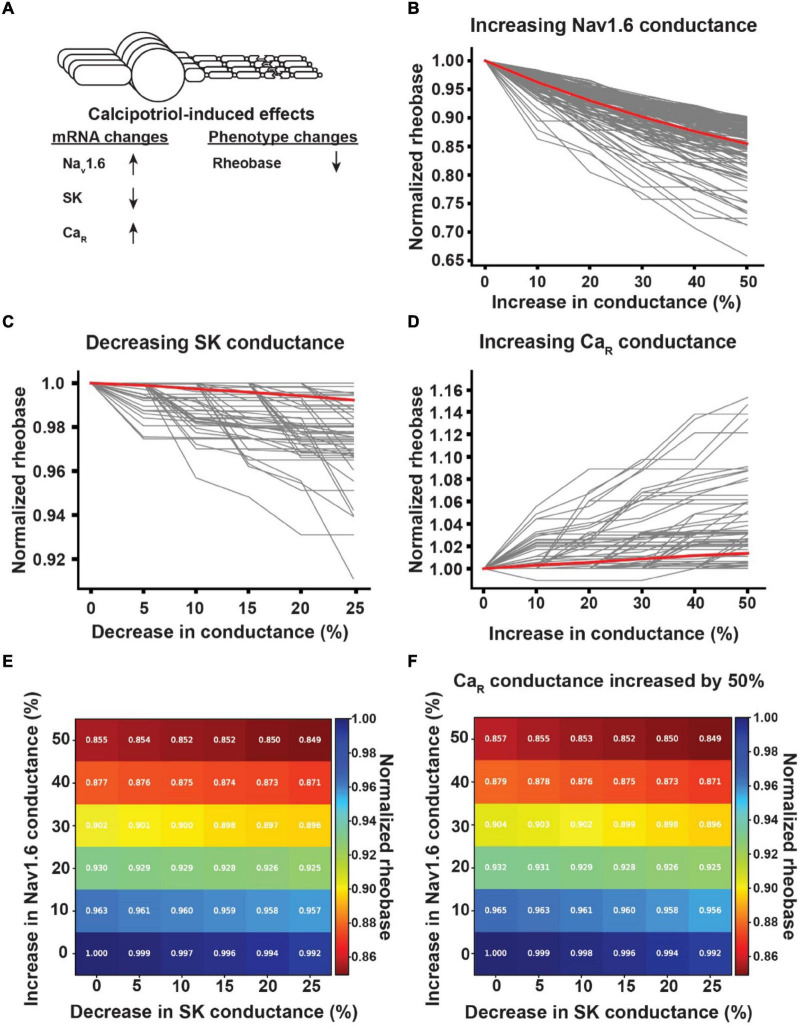
Effect of calcipotriol-induced changes in ion channel expression on the excitability of Ucn3^+^ neurons. **(A)** Calcipotriol-induced effects on Ucn3^+^ neurons. **(B)** Effect of increasing Nav1.6 conductance on Ucn3^+^ model neuron rheobases. **(C)** Effect of decreasing SK conductance on Ucn3^+^ model neuron rheobases. **(D)** Effect of increasing Ca2.3-containing R-type Ca channel (Ca_R_) conductance on Ucn3^+^ model neuron rheobases. **(E)** Effect of simultaneously increasing Nav1.6 conductance and decreasing SK conductance on the average rheobase of the Ucn3^+^ model neuron population. **(F)** Effect of simultaneously increasing Nav1.6 conductance, decreasing SK conductance, and increasing Ca_R_ conductance by 50% on the average rheobase of the Ucn3^+^ model neuron population.

In calcipotriol-treated mice, the rheobase of Ucn3^+^ neurons decreased, the expression levels of Nav1.6 and Cav2.3 increased, and the expression level of SK decreased (Figure 6A). When we increased the Nav1.6 conductance in Ucn3^+^ model neurons by 50%, the Ucn3^+^ model neurons experienced between a 9.8 and 34.2% reduction in rheobase, with an average reduction in rheobase of 14.5% (Figure 6B). When we decreased the SK conductance in Ucn3^+^ model neurons by 25%, the Ucn3^+^ model neurons experienced between a 0 and 8.9% reduction in rheobase, with an average reduction in rheobase of 0.8% (Figure 6C). When we increased the Cav2.3 conductance in Ucn3^+^ model neurons by 50%, the Ucn3^+^ model neurons experienced between a 0 and 15.3% increase in rheobase, with an average increase in rheobase of 1.4% (Figure 6D). We next examined how varying the conductances of multiple ion channels simultaneously affected the rheobase of Ucn3^+^ model neurons (Figures 6E,F). Generally, the rheobase was most significantly affected by increasing the conductance of Nav1.6. When we increased the Nav1.6 conductance by 50% and decreased the SK conductance by 25%, Ucn3^+^ model neurons experienced between a 9.8 and 41.5% reduction in rheobase, with an average reduction in rheobase of 15.1% (Figure 6E). When we additionally increased the Ca2.3 conductance by 50%, Ucn3^+^ model neurons experienced a reduction between 9.8 and 38.3% in rheobase, with an average reduction of 15.1% in rheobase (Figure 6F). The average reduction in rheobase by maximally varying the conductance of multiple ion channels (i.e., 15.1%) was comparable to the average reduction in rheobase by only increasing Nav1.6 conductance by 50% alone (i.e., 14.5%).

**FIGURE 7:**
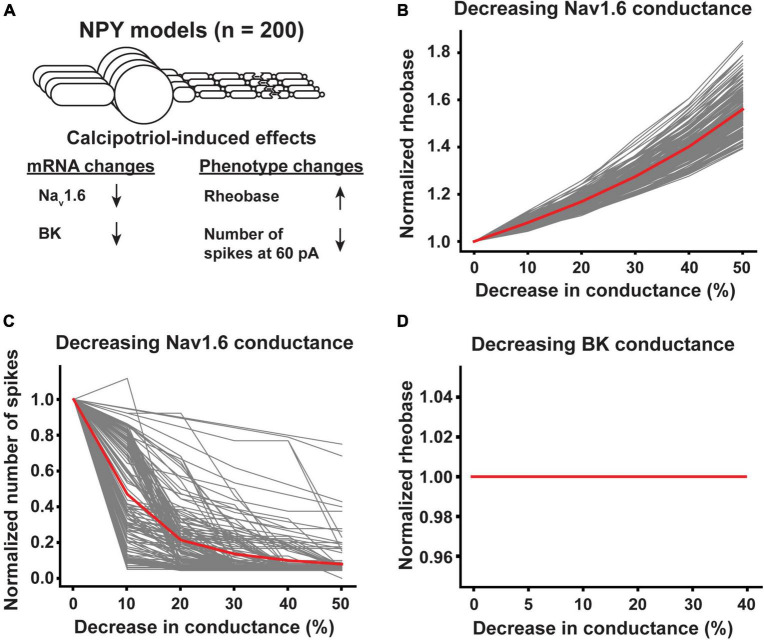
Effect of calcipotriol-induced changes in ion channel expression on the excitability of NPY^+^ neurons. **(A)** Calcipotriol-induced effects on NPY^+^ neurons. **(B)** Effect of decreasing Nav1.6 conductance on NPY^+^ model neuron rheobases. **(C)** Effect of decreasing Nav1.6 conductance on NPY^+^ model neuron numbers of spikes generated in response to 60 pA current injection. **(D)** Effect of decreasing BK conductance on NPY^+^ model neuron rheobases.

By contrast, the rheobase of NPY^+^ neurons increased, the number of spikes generated in response to 60 pA current-clamp stimulation decreased, and the expression of Nav1.6 and BK decreased in calcipotriol-treated mice (Figure 7A). When we decreased the Nav1.6 conductance in NPY^+^ model neurons by 50%, NPY^+^ model neurons experienced between a 39.3 and 84.9% increase in rheobase, with an average increase in rheobase of 56.0% (Figure 7B). In addition, a 50% decrease in Nav1.6 conductance in NPY^+^ model neurons produced between a 25.0 and 100.0% reduction in the number of spikes generated during 60 pA current-clamp stimulation, with an average decrease of 92.1% (Figure 7C). Decreasing BK conductance by up to 40% had no effect on rheobase in any NPY^+^ model neuron (Figure 7D) or the number of spikes generated during 60 pA current-clamp stimulation (data not shown). We did not vary the conductance of Nav1.6 and BK simultaneously in our NPY^+^ model neurons because changing BK conductance alone did not produce an effect on the excitability metrics. Our modeling results indicate that Nav1.6 may be the major component to alter excitability in both Ucn3^+^ and NPY^+^ neurons under chronic itch conditions.

### Effect of Ion Channel Conductance on Gating Mechanical Itch

**FIGURE 8:**
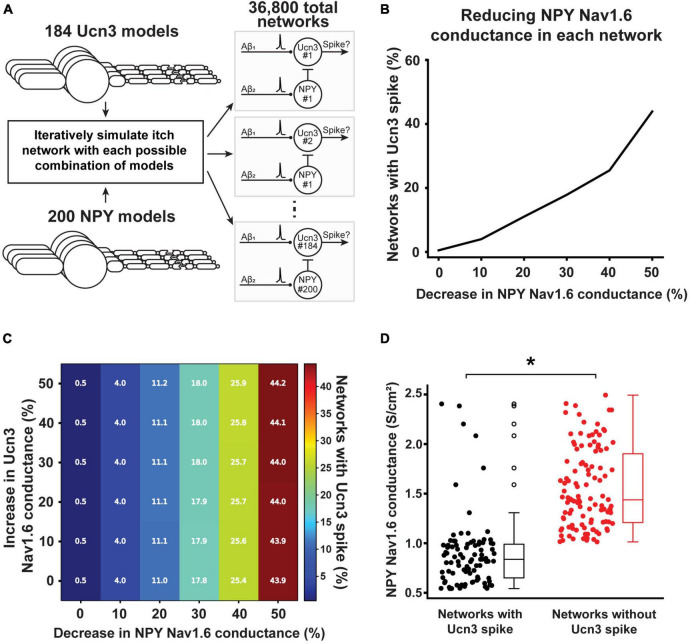
Effect of calcipotriol-induced changes in ion channel expression on gating mechanical itch. **(A)** Simulating every permutation of the mechanical itch network with our 184 Ucn3^+^ model neurons and 200 NPY^+^ model neurons. In total, we simulated 36,800 distinct networks. **(B)** Effect of decreasing Nav1.6 conductance in the NPY^+^ model neuron on the percentage of networks producing a spike in its Ucn3^+^ model neuron. **(C)** Effect of simultaneously decreasing Nav1.6 conductance in the NPY^+^ model neuron and increasing Nav1.6 conductance in the Ucn3^+^ model neuron on the percentage of networks producing a spike in its Ucn3^+^ model neuron. **(D)** Comparing the baseline Nav1.6 conductance in NPY^+^ model neurons present in networks that produced a spike in the Ucn3^+^ model neuron (black) and networks that did not produce a spike in the Ucn3^+^ model neuron (red). *p < 0.001. Mann-Whitney *U* rank test.

We next used computational modeling to examine whether altered Nav1.6 conductance is the key mechanism to sensitize the mechanical itch circuit in the spinal cord under chronic conditions. In our computational models, we investigated which changes of Nav1.6 conductance in Ucn3^+^ and NPY^+^ neurons lead to AP generation in Ucn3^+^ neurons in response to physiological Aβ-fiber input. We simulated each permutation of the 200 NPY^+^ model neurons and 184 Ucn3^+^ model neurons for a total of 36,800 individual networks (Figure 8A). At baseline (i.e., without changing the Nav1.6 conductance in any Ucn3^+^ or NPY^+^ model neurons), 0.5% of the simulated networks (184 out of 36,800 total networks) had a Ucn3^+^ model neuron that generated an AP in response to physiological Aβ-fiber input. We then simulated how changes in the Nav1.6 conductance in NPY^+^ neurons affected Aβ-evoked AP generation in Ucn3^+^ neurons. Decreasing Nav1.6 conductance by 50% in NPY^+^ model neurons resulted in 43.9% of simulated networks (16,148 out of 36,800 total networks) having a Ucn3^+^ model neuron generating an AP in response to physiological Aβ-fiber input (Figure 8B). Next, we examined how concurrent changes in Nav1.6 conductance in both Ucn3^+^ and NPY^+^ model neurons affected Aβ-evoked AP generation in Ucn3^+^ neurons (Figure 8C). Simultaneously decreasing Nav1.6 conductance in NPY^+^ model neurons by 50% and increasing Nav1.6 conductance in Ucn3^+^ model neurons by 50% resulted in 44.2% of simulated networks (16,258 out of 36,800 total networks) producing an AP in the Ucn3^+^ model neuron (Figure 8C), slightly increasing the network excitability compared with the results in Figure 8B. Lastly, we examined if Nav1.6 conductance was different between neurons in the mechanical itch circuit with an open gate (Aβ inputs can generate an AP in the Ucn3^+^ model neuron) compared to a closed gate (Aβ inputs cannot generate an AP in the Ucn3^+^ model neuron). We found that NPY^+^ model neurons in the microcircuits that produced an Aβ-evoked AP in the Ucn3^+^ neuron had significantly lower Nav1.6 conductance than the microcircuits that did not produce an Aβ-evoked AP in the Ucn3^+^ neuron (*p < 0.001. Mann-Whitney *U* rank test; Figure 8D). Our results suggest that decreased Nav1.6 conductance in NPY^+^ neurons may play the major role in sensitizing the mechanical itch circuit under chronic itch conditions.

## Discussion

Our experimental recordings demonstrated that Ucn3^+^ mechanical itch transmission neurons in the dorsal spinal cord become more excitable under chronic itch conditions, while NPY^+^ inhibitory gating neurons in the dorsal spinal cord become less excitable. Furthermore, single-cell qRT-PCR experiments showed that the expression levels of Nav1.6 and Cav2.3 channels increased in Ucn3^+^ neurons in chronic itch mice, while the expression level of SK3 channels decreased. In NPY^+^ neurons, the expression levels of Nav1.6 and BK channels decreased in chronic itch mice. Computational modeling data showed that reduced Nav1.6 conductance in NPY^+^ neurons might be a key contributor to excite Ucn3^+^ neurons and develop mechanical itch sensitization.

### Decreased Nav1.6 Conductance in Neuropeptide Y-Positive Neurons Opens the Mechanical Itch Gate in Chronic Itch

Studies on acute itch have led to the identification of molecular and cellular candidates to treat chronic itch. Sensitization of acute itch circuits is one of the key mechanisms underlying the transition from acute itch to chronic itch (Bautista et al., 2014; LaMotte et al., 2014; Cevikbas and Lerner, 2020; Lay and Dong, 2020). In the periphery, many studies have focused on the communication between skin cells and immune cells that sensitize itch-selective primary sensory neurons (Luo et al., 2015; Mack and Kim, 2018; Donnelly et al., 2020). In the spinal cord, central sensitization of NK1R^+^ neurons accounts for persistent spontaneous itch under chronic itch conditions (Akiyama et al., 2015; Sheahan et al., 2020). Alternatively, disinhibition of itch transmission pathways in the spinal cord can also contribute to chronic itch (Braz et al., 2014). This study indicates that disinhibition of Ucn3^+^ neurons *via* reducing excitability of NPY^+^ neurons might play an essential role to sensitize mechanical itch circuits in the spinal cord, consistent with previous studies that ablation of NPY^+^ interneurons in the dorsal spinal cord can develop mechanical itch sensitization and persistent spontaneous itch in mice (Bourane et al., 2015; Pan et al., 2019). Interestingly, our results suggest that a reduction in Nav1.6 conductance in NPY^+^ neurons has the greatest effect on Ucn3^+^ neurons generating a spike in response to physiological Aβ-fiber input. Concurrently varying Nav1.6 conductance in both Ucn3^+^ and NPY^+^ model neurons produced only slight changes in the percentage of networks with a Ucn3^+^ neuron spike compared to varying Nav1.6 conductance in NPY^+^ model neurons alone. Low-threshold Nav1.6 channels preferentially accumulate at the distal axon initial segment and promote AP initiation, which plays a significant role in regulating neuronal repetitive discharge behavior (Hu et al., 2009). Therefore, decreased expression of Nav1.6 channels in NPY^+^ neurons might be a key contributor to the disinhibition underlying the development of chronic itch. Novel therapeutics aimed toward upregulating the expression of Nav1.6 channel or augmenting the activity of existing Nav1.6 channels in NPY^+^ mechanical itch gating neurons may be worth exploring for managing chronic itch conditions.

### Modeling Populations of Biophysically Distinct Neurons

Typically, multicompartment neuron models are parameterized (e.g., maximal ion channel conductances) such that the singular model reproduces the means of experimentally measured properties (e.g., rheobase). However, inherent biological variability between neurons (e.g., varying expression levels of different ion channels) suggests that neurons parameterized to reproduce the means of experimental data may not represent an actual neuron from the sampled population (Marder and Taylor, 2011). Furthermore, disparate combinations of maximal ion channel conductances can produce similar model cell behaviors (e.g., similar firing patterns in response to current-clamp stimulation) (Marder and Goaillard, 2006; Marder and Taylor, 2011). It is unclear if similarly behaving but biophysically distinct cells are differentially affected by the physiological changes observed during pathogenesis. It is also unclear if such pathological changes differentially affect the activity of networks comprised of biophysically distinct cells. Understanding the effects of biological variability on the behavior of single neurons and neural networks during pathogenesis is critical to understanding which physiological parameters contribute to disease phenotypes across neuronal populations.

In this work, we modeled populations of Ucn3^+^ and NPY^+^ model neurons. We sought to understand how changes in ion channel expression affected both the excitability of each single-cell model and the output of the mechanical itch network when constructed with different permutations of cell combinations. In general, many individual model neurons responded similarly to changes in ion channel conductance. However, there were two instances where individual cell models responded differently to a change in conductance than the rest of the population. One Ucn3^+^ model experienced a reduction in rheobase in response to a 10–30% increase in Cav2.3 conductance while all other cells only experienced increases in rheobase (Figure 6D). Similarly, one NPY^+^ model neuron experienced an increase in the number of spikes generated during 60 pA current injection when Nav1.6 conductance was decreased by 10% while all other cells experienced only decreases (Figure 7C). Though these are only two examples out of a large number of cells, these examples highlight that simulating many distinct neurons could reveal subpopulations of cells that respond differently to the same physiological stimulus.

### Limitations

Our model populations are currently simulating the most excitable subset of our experimentally measured Ucn3^+^ and NPY^+^ neurons in both control and chronic itch groups. There may be significant ionic current components present in Ucn3^+^ and NPY^+^ neurons that are not currently included in our neuron models. Furthermore, measuring changes in mRNA expression is not a direct measurement of functional protein insertion into the cell membrane. Novel methods for measuring the spatiotemporal profiles of ion channel insertion into neural membranes could provide crucial data with which to parameterize multicompartment neuron models like those used in this study.

## Conclusion

In this study, we used a combination of electrophysiological recording, single-cell qRT-PCR and computational modeling to reveal that reduced Nav1.6 conductance in spinal NPY^+^ inhibitory interneurons may play a major role in sensitizing mechanical itch pathways under chronic itch conditions. Such a pairing of experimental measurement and model design provides insight into the biophysical mechanisms of disease pathogenesis and assists the design of novel targeted therapeutics.

## Data Availability Statement

The raw data supporting the conclusions of this article will be made available by the authors, without undue reservation, to any qualified researcher.

## Ethics Statement

The animal study was reviewed and approved by the Institutional Animal Care and Use Committee at the University of Michigan.

## Author Contributions

BD and SL conceptualized the study. HL performed the electrophysiological recording. HL, DM, and BS performed qRT-PCR experiments. RG and EM performed the computational modeling. HL and RG analyzed the data. BD, SL, HL, and RG wrote the manuscript. All authors contributed to the article and approved the submitted version.

## Conflict of Interest

The authors declare that the research was conducted in the absence of any commercial or financial relationships that could be construed as a potential conflict of interest.

## Publisher’s Note

All claims expressed in this article are solely those of the authors and do not necessarily represent those of their affiliated organizations, or those of the publisher, the editors and the reviewers. Any product that may be evaluated in this article, or claim that may be made by its manufacturer, is not guaranteed or endorsed by the publisher.

## Footnotes

1
https://emcee.readthedocs.io
